# Facing Mpox (Former Monkeypox) in Latin America: The Example of Peru and Its Vulnerable Healthcare System

**DOI:** 10.3390/vaccines11010010

**Published:** 2022-12-20

**Authors:** Ali Al-kassab-Córdova, Juan R. Ulloque-Badaracco, Vicente A. Benites-Zapata, Ranjit Sah, Alfonso J. Rodriguez-Morales

**Affiliations:** 1 Centro de Excelencia en Estudios Económicos y Sociales en Salud, Universidad San Ignacio de Loyola, Lima 15024, Peru; 2 Facultad de Ciencias de la Salud, Universidad Peruana de Ciencias Aplicadas, Lima 15067, Peru; 3 Unidad de Investigación para la Generación y Síntesis de Evidencias en Salud, Universidad San Ignacio de Loyola, Lima 15024, Peru; 4 Tribhuvan University Teaching Hospital, Institute of Medicine, Kathmandu 44600, Nepal; 5 Department of Global Health and Clinical Research, Harvard Medical School, Boston, MA 02115, USA; 6 Dr. D.Y Patil Medical College, Hospital and Research Center, Dr. D.Y. Patil Vidyapeeth, Pune 411018, Maharashtra, India; 7 Grupo de Investigación Biomedicina, Faculty of Medicine, Fundación Universitaria Autónoma de las Américas—Institución Universitaria Visión de las Américas, Pereira 660003, Risaralda, Colombia; 8 Master of Clinical Epidemiology and Biostatistics, Universidad Científica del Sur, Lima 4861, Peru; 9 Gilbert and Rose-Marie Chagoury School of Medicine, Lebanese American University, Beirut P.O. Box 36, Lebanon

The new outbreak of monkeypox, a viral zoonotic disease, has affected more than 82,500 people and at least 110 countries worldwide as of 14 December 2022, with 81,580 people affected in 103 non-endemic areas of Africa [1]. The monkeypox virus is a DNA Orthopoxvirus that is part of the variola virus family (Poxviridae), which causes smallpox. Symptoms of monkeypox, however, are milder and rarely life-threatening. Recently, after a series of consultations with global experts, the World Health Organization (WHO) proposed “Mpox” as a new preferable term, replacing monkeypox [2]. The arrival of monkeypox to multiple regions, including Latin America, an area whose countries are primarily of low- and middle-income and that were severely affected by the SARS-CoV-2/COVID-19 pandemic during 2020–2022, has raised the necessity of further preparedness to deal with a new epidemic, which was declared a Public Health Emergency of International Concern (PHEIC) by the WHO, on 23 July 2022 [3]. However, budget cuts and the structural deficiencies of the Latin American health systems pose additional challenges to dealing with this threat.

On 9 August 2022, the first case of monkeypox in Peru was reported in an 8-year-old boy; since then, there has been a constant increase in cases in that country, as well as in other Latin America countries. As of 24 November, 3466 cases distributed throughout 20 of the 24 regions of Peru have been reported. No deaths, officially, have been reported [4]. This has placed Peru as the Latin American country with the third-most cases, after Brazil and Colombia, but the first in number of cases per population (101.15 cases/1,000,000 pop.) (Figure 1). The upward trend in cases, alongside the fragmentation and segmentation of the healthcare system, poses a threat to outbreak control at a national level. In addition, a recent study found that Peruvian health professionals had insufficient knowledge of the aetiology, symptoms, treatment, and prevention of monkeypox [5]. Nevertheless, despite this scenario, a few strategies have been implemented.

The Peruvian Ministry of Health (MINSA, from the Spanish acronym) has created some promotional and preventive strategies to ameliorate the impact of monkeypox. Accordingly, all suspected cases must undergo 21-day isolation from the appearance of symptoms or until the disappearance of the scabs. Additionally, any close contacts should be followed up for 21 days. Moreover, itinerant teams (composed of health professionals, peer educators, or community agents) should be deployed to conduct promotional activities on preventive measures in physical and virtual spaces [6]. Nevertheless, the strategies mentioned above are unsustainable without an extensive vaccination campaign. 

The vaccines arrived more than two months after the first reported monkeypox case. According to official data, only 5600 and 4200 JYNNEOS^®^ vaccines arrived in October and November 2022, respectively. These quantities are deficient compared to the population to be vaccinated, even requiring priority according to risk. Likewise, conducting comprehensive vaccination campaigns with an informative approach is paramount, as vaccine acceptance in the general population is pretty low [7]. Due to the lack of vaccines, the most vulnerable groups should be prioritized, such as the LGBTI+ community.

Several actions are urgently required to address the monkeypox outbreak in the Peruvian setting and, all in all, in Latin America. First, it is imperative to perform educational activities, with an emphasis on preventive measures, aimed at health professionals and the general population. Second, free detection campaigns must be carried out at the national level but prioritizing vulnerable groups. Third, even though the access to vaccines and treatment is still limited in the region, policymakers must ensure their availability in the short-term. Fourth, we encourage strengthening epidemiological surveillance systems to provide updated data. As well, due to the low acceptance of vaccination [7], it should be monitored. Fifth, governmental standardized guidelines should be promptly published and constantly updated. The prompt implementation of these measures could lessen the impact of the outbreak.

The example of Peru is similar to other affected countries in Latin America [7]. Due to the many shortcomings of the Peruvian health system, it is vulnerable to outbreaks and other public health threats and unable to respond adequately to them. Therefore, it deserves more attention from local, national, and regional health authorities because, although this is not a monkeypox pandemic, there is a clear impact on risk groups that may even evolve into fatal outcomes [8].

Mpox is a good example demonstrating the need for more preparedness for emerging disease epidemics. Countries, especially those which are more vulnerable, need to develop more comprehensive ways to address such biological threats, include preparedness committees, and protocols. Healthcare facilities should be also trained for the event of emerging diseases, such as Mpox.

## Figures and Tables

**Figure 1:**
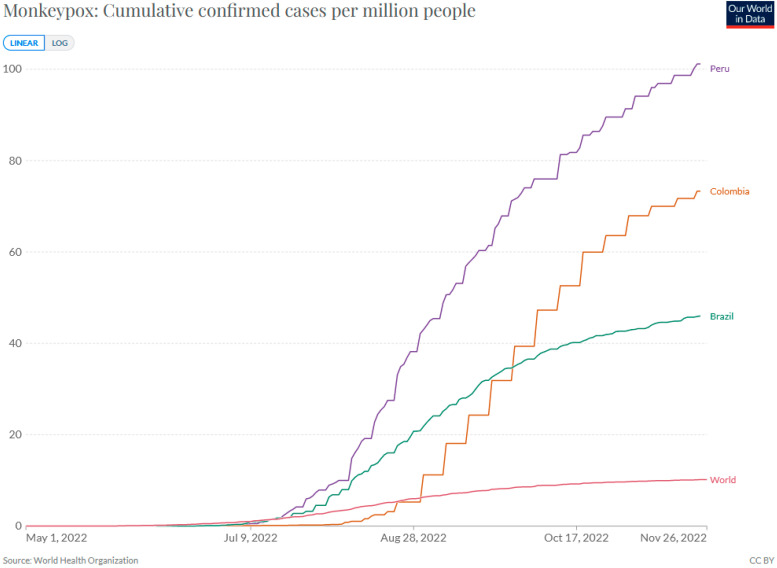
Cumulative rate of cases per 1,000,000 people. Source: https://ourworldindata.org/monkeypox (accessed on 26 November 2022).

## Data Availability

Data sharing not applicable.
